# Elucidation of the genetic basis of variation for stem strength characteristics in bread wheat by Associative Transcriptomics

**DOI:** 10.1186/s12864-016-2775-2

**Published:** 2016-07-16

**Authors:** Charlotte N. Miller, Andrea L. Harper, Martin Trick, Peter Werner, Keith Waldron, Ian Bancroft

**Affiliations:** John Innes Centre, Norwich Research Park, Norwich, NR4 7UH UK; KWS UK Ltd, 56 Church Street, Thriplow, Hertfordshire SG8 7RE UK; Institute of Food Research, Norwich Research Park, Norwich, NR4 7UH UK; Department of Biology, University of York, York, YO10 5DD UK

**Keywords:** Modulus of Rupture, lodging, Associative Transcriptomics, Xylan acetylation, COP9 signalosome, Auxin

## Abstract

**Background:**

The current approach to reducing the tendency for wheat grown under high fertilizer conditions to collapse (lodge) under the weight of its grain is based on reducing stem height via the introduction of *Rht* genes. However, these reduce the yield of straw (itself an important commodity) and introduce other undesirable characteristics. Identification of alternative height-control loci is therefore of key interest. In addition, the improvement of stem mechanical strength provides a further way through which lodging can be reduced.

**Results:**

To investigate the prospects for genetic alternatives to *Rht*, we assessed variation for plant height and stem strength properties in a training genetic diversity panel of 100 wheat accessions fixed for *Rht*. Using mRNAseq data derived from RNA purified from leaves, functional genotypes were developed for the panel comprising 42,066 Single Nucleotide Polymorphism (SNP) markers and 94,060 Gene Expression Markers (GEMs). In the first application in wheat of the recently-developed method of Associative Transcriptomics, we identified associations between trait variation and both SNPs and GEMs. Analysis of marker-trait associations revealed candidates for the causative genes underlying the trait variation, implicating xylan acetylation and the COP9 signalosome as contributing to stem strength and auxin in the control of the observed variation for plant height. Predictive capabilities of key markers for stem strength were validated using a test genetic diversity panel of 30 further wheat accessions.

**Conclusions:**

This work illustrates the power of Associative Transcriptomics for the exploration of complex traits of high agronomic importance in wheat. The careful selection of genotypes included in the analysis, allowed for high resolution mapping of novel trait-controlling loci in this staple crop. The use of Gene Expression markers coupled with the more traditional sequence-based markers, provides the power required to understand the biological context of the marker-trait associations observed. This not only adds to the wealth of knowledge that we strive to accumulate regarding gene function and plant adaptation, but also provides breeders with the information required to make more informed decisions regarding the potential consequences of incorporating the use of particular markers into future breeding programmes.

**Electronic supplementary material:**

The online version of this article (doi:10.1186/s12864-016-2775-2) contains supplementary material, which is available to authorized users.

## Background

Lodging is defined as the permanent displacement of a crop from its usually vertical growth habit. This phenomenon may be divided into two main categories: lodging caused by anchorage failure, or root lodging; and lodging caused by stem mechanical failure, also known as brackling or stem lodging. Lodging is a complex trait, influenced by environmental, agronomic and genetic factors and continues to be a widespread problem in wheat grown worldwide. In years where lodging is particularly severe, yield losses as great as 80 % can be expected [1].

Previous efforts to reduce the occurrence of lodging in wheat have centred on reducing the height of plants through incorporation of semi-dwarfing alleles into accessions and the use of plant growth regulators (PGR). The most common semi-dwarfing genes found in modern wheat accessions are the GA-insensitive *Rht-B1* and *Rht-D1*, which markedly increased the yield potential of wheat following their introduction [2]. However, these genes may not be beneficial under some environmental conditions, and efforts to identify other semi-dwarfing genes with different physiological functions are ongoing. Another potential strategy is to breed accessions with increased mechanical strength in the plant stems. While stem mechanical strength is considered an important agronomic trait, few studies have focused on the identification of genetic markers for this trait which may be utilised in marker-assisted breeding. Furthermore, the few mapping studies that have been conducted with this aim have been limited by low marker density and mapping resolution through the utilisation of the traditional bi-parental cross, QTL analysis approach [3, 4].

In recent years we have seen the successful application of GWAS in a number of different plant species [5–7]. This method makes use of historical recombination events which, when coupled with high marker density, provides increased mapping resolution. Furthermore, recent advances have expanded this powerful mapping approach to combine the exploration of marker variation at both the sequence and gene expression level in a method termed, Associative Transcriptomics (AT) [8]. The ordered transcriptome resource necessary for implementation of AT in hexaploid wheat has been established [9].

Our aim was to explore the variation available in European wheat breeding material for both height and stem mechanical strength, and in the first application of AT in wheat, to identify molecular markers associated with this variation. This will provide breeders with both insights into the bases of variation for these traits and molecular markers to underpin marker-assisted breeding of wheat accessions with improved lodging resistance.

## Methods

### Plant material and phenotyping

**Fig. 1:**
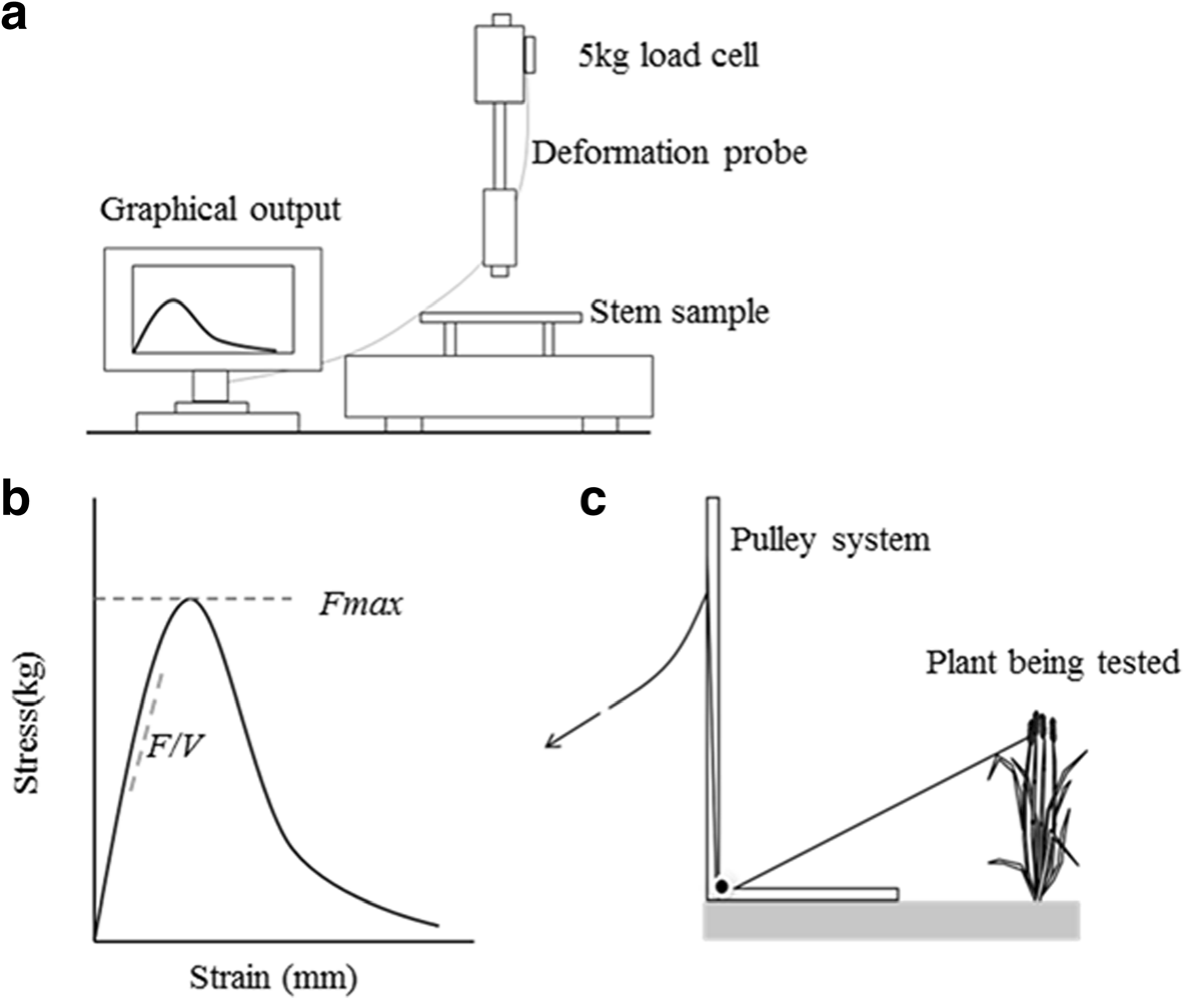
Apparatus used for assessment of stem mechanical strength in wheat. A lab-based three-point bend test setup (**a**) allowed for the absolute strength traits, Fmax (the resistance of the stem sample to break under load) and F/V (the resistance of the stem sample to bend elastically), to be obtained (**b**). A field-based stem lodging risk measure was obtained using a pulley device (**c**)

A panel of 100 European accessions of hexaploid bread wheat, *Triticum aestivum* (Additional file 1), was grown at a single site (KWS, Thriplow, UK) across 2 years (2011 and 2012). In 2012, prior to harvesting, a subset of accessions was screened for stem lodging risk. Using a pulley system attached to the base of the ear of each plant tested (Fig. 1c), stems were pulled through a reproducible arc path (to ground level), a similar motion to that which would be induced by wind or heavy rainfall. Following this, any stem mechanical failure induced by the bending of the stem was recorded. Stems found to suffer stem breakage were scored with a “1” and those for which no mechanical failure was observed were scored with a “0”. This experiment was performed for 6 plants per accession and a mean “breakage score” determined.

Harvesting of material was carried out by hand, cutting the stems as close to the soil as possible using secateurs. Ten plants were harvested for each accession in 2011 and five plants per accession in 2012. Prior to further processing, all plants were dried thoroughly at room temperature. Any plants showing signs of stem tissue damage were excluded from the study. To allow for an in-depth analysis of the relationship between plant morphology, stem structure and stem mechanical strength, the following measurements were determined for each plant harvested: Plant height; main stem (determined as the tallest) weight and threshed weight; length of the second internode (from plant base) and stem width (measured using digital callipers at the midway point of the second internode). A 5 cm section was then removed from the 2nd internode of the main stem using a scalpel. The basal end of this section was marked using a permanent marker pen. To obtain stem cross-sectional measurements (required for the later calculation of stem second moment of area (*I*)), the transverse of the marked stem end was photographed. All images were later analysed using the digital analysis software Sigma Scan (Stystat Software Inc., San Jose, USA), allowing the following cross-sectional measurements to be determined: whole stem area (used in the later calculation of *D2*); stem hollow area (used in the later calculation of *d1a*); the area of stem parenchyma and the thickness of the stem outer cortex. Following these initial measurements, all samples were stored at 55 % relative humidity at 23 °C for a minimum of 2 days in a silica chamber to ensure equilibration of moisture content between samples.

Mechanical testing of the material was carried out using a Texture Analyser (TA) (Analyser (TA-XT2®- Stable Microsystems, Godalming, UK) with a three-point bend test setup (Institute of Food Research, Norwich, UK) (Fig. 1a). These methods were adapted from Kern et al. [10]. The TA was fitted with a load cell with maximum loading capacity of 5 kg. The support stands were set at 2.5 cm apart (across which the 5 cm stem sample was placed) and the testing probe was set to move at a constant speed of 2 mm/s. The TA, connected to a computer, produces a real-time graphical output, representing the mechanical profile of the stem sample being tested. From this graph, *Fmax*, the absolute resistance of the stem sample to break under-load, and *F/V*, the resistance of the stem sample to bend elastically, were obtained (Fig. 1b). These are ‘absolute strength measures’, being the result of a combination of both strength due to structure and material strength. These absolute measures of strength, together with the stem sample second moment of area (*I*) (Eq. (1)), were used in calculating the material strength of the stem samples: the Modulus of Rupture (*MOR*), describing the resistance of the stem material to break under-load (Eq. (3)) and the Modulus of Elasticity (*MOE*) describing the resistance of the stem material to bend elastically (Eq. (2)).

### Equations

1 $$ I=\pi \left(\mathrm{D}{2}^4-\mathrm{D}1{\mathrm{a}}^4\right)/64 $$

Where:

*D2*: = diameter of whole stem calculated from stem cross-sectional area
*D1a*: = diameter of stem hollow calculated from stem hollow area

2 $$ MOR=\left(\mathrm{Fmax}*\mathrm{a}*\mathrm{D}2\right)/I $$

3 $$ MOE=\left(\mathrm{F}/\mathrm{V}\right)*\left({\mathrm{a}}^2/12\right)*\left(3\mathrm{L}-4\mathrm{a}\right)/I $$

Where:

*L*: = the length of the stem sample between the two supports
*a*: = L/2

### Statistical analysis of data

Following the assessment of year by year interactions, traits were assessed for significant genotypic variation and REML-predicted means calculated for use in the subsequent correlation (Genstat 15th edition) and Associative Transcriptomics analyses. These statistical analyses were carried out using Genstat 15th edition (VSN International, Hemel Hempstead, UK).

### mRNA-seq and marker scoring

For the mRNA-seq, second true leaves from each of four plant replicates per accession were harvested approximately 14 days after pricking out (21 day after sowing) as close to the midpoint of the light period as possible, pooled and immediately frozen in liquid nitrogen. Samples were extracted using the Omega Biotek EZNA Plant RNA Kit according to manufacturer’s instructions.

Transcriptome sequence data was then obtained for each of the 100 wheat accessions included in the training panel. This was achieved using Illumina transcriptome sequencing (mRNA-seq). Illumina sequencing, quality checking and processing were conducted as described previously [11] except that, for SNP calling and transcript quantification, 100 base reads obtained from the HiSeq platform were trimmed in order to retain comparability with 80 base reads generated on GAIIx instruments, and capped at 35 million reads to maintain comparable read depth. Maq was used for mapping with default parameters, meaning that reads with no more than two mismatches with summed Q ≥ 70 were mapped.

The alignment of these reads for SNP detection was facilitated by the development of a reference sequence, as described previously [9]. Briefly, the reference sequence was generated based on *de novo* transcriptome assemblies of *Triticum urartu*, *Aegilops speltoides* and *Aegilops tauschii* (representing the A, B and D genomes, respectively) generated using the Trinity assembly package [12]. The B genome was further improved by “curing” [13] using sequence information from the tetraploid *T. turgidum* ssp. *dicoccoides*, which more closely represents the B genome in hexaploid wheat. This resulted in a reference transcriptome sequence comprising 105,069, 132,363 and 85,296 transcript assemblies for the A, B and D genomes respectively. Based on linkage map information and conserved synteny between wheat and *Brachypodium distachyon*, these assemblies were arranged into their hypothetical gene order, providing a set of pseudomolecules [9]. Based on sequence similarity to Brachypodium, rice, sorghum and Maize, these pseudomolecules were annotated with probable gene functions. The A, B and D reference assemblies were sufficiently distinct to enable reads to be aligned in a genome-specific manner.

The alignment of the diversity panel mRNA-seq reads to this reference sequence enabled the detection of 42,066 SNP markers. SNP-calling was conducted essentially as described previously for *Brassica napus*, with read mapping and SNP calls made for each accession using Maq and Maq.pl commands, before integrating calls across the panel using the Perl script combiner.pl [11]. Simple SNPs were called by the meta-analysis of alignments against the Trinity unigene reference from mRNA-Seq reads obtained from each of 100 bread wheat lines. SNP positions were excluded from further analysis if more than two alleles were detected across the accessions, and a noise threshold of 0.15 was employed to reduce false SNP calls due to sequencing errors.

In addition, quantification of transcript abundance (as reads per kb per million aligned reads; RPKM) provided a measure of expression for each transcript assembly. This provided the information required to explore any relationships between gene expression and the trait of interest in what has been termed a Gene Expression Marker (GEM) analysis [8].

### SNP marker analysis

SNP-based association analysis was performed using 12,456 SNPs (following the removal of SNPs present at minor allele frequency <5 %). The results were assessed visually by plotting the obtained *P* values (as –log_10_*P*) in pseudomolecule order.

The filtered SNP dataset was used to construct a kinship matrix using the software TASSEL V3.0 [14]. In addition, broad-scale population structure was assessed using the Bayesian clustering method, STRUCTURE [15]. Based on the SNP data, this method was used to identify ancestral groups to which the different accessions could be apportioned. The SNP data was processed using an admixture model with independent allele frequencies. To allow for likelihood estimates of a range of ancestral populations to be made, the model was set to run with hypothetical population (K) estimates of 1 to 5. The SNP data was processed for each value of K three times with a burn-in length of 100,000. This was followed by 100,000 iterations of the Monte Carlo Markov Chain algorithm. To allow for a more accurate estimate of K, the results obtained from STRUCTURE were further analysed using the methods described by Evanno et al. [16] allowing for the assessment of variance between iterations. The output of the STRUCTURE analysis was used as a Q matrix (Additional file 2) for the subsequent Associative Transcriptomics analyses. The trait data, SNP data, Q matrix and kinship matrix were incorporated into a Mixed Linear Model (MLM) algorithm performed using TASSEL.

### Gene Expression Marker (GEM) analysis

Following the quantification and normalisation of the transcript levels as reads per kb per million aligned reads (RPKM), and the filtering of transcripts with an RPKM of less than 0.4 across accessions, linear regression was performed. This analysis made use of 94,060 GEM markers. By fitting the RPKM values for each unigene as the dependant variable and the trait data as the independent variable, it was possible to assess the relationship between this measure of gene expression and the traits. The results were explored visually by plotting the obtained *P* values (as –log_10_*P*) in pseudomolecule order. The scripts used in this analysis were developed in R (cran.r-project.org).

### Validation of markers

Leaf material was collected from a test panel of 96 hexaploid wheat accessions grown as part of the WAGTAIL panel (a diversity panel developed for the BBSRC LINK project “Wheat Association Genetics for Trait Analysis and Improved Lineages” (BB/J002607/1)) at KWS, Thriplow in 2013. DNA was extracted according to an adapted method of that described by Pallotta et al. [17]. Genome specific primers were designed for each of the marker loci analysed. All marker assays were first tested on wheat accessions of known genotype (a subset of the Associative Transcriptomics panel). Following confirmation that the marker assays were able to effectively screen for the target variation, they were further used to genotype the 96 WAGTAIL accessions. All genotyping was performed using AMPLITAQ Gold polymerase (250 u – Life Technologies Ltd (Invitrogen Division, Paisley, UK)). Prior to sequencing, PCR reactions were purified using the ExoSAP protocol [18]. Following this, sequencing reactions were set up in 0.2 ml tubes according to a revised protocol from BigDye V3.1 terminator cycle sequencing kit [19]. All PCR and sequencing reactions were performed using a G-Storm GS1 thermal cycler (Somerton, UK). Capillary sequencing was performed by GATC Biotech AG, Germany and all sequencing trace files obtained were analysed using Contig Express (Vector NTI advance® 11.5.2, Paisley, UK).

Following genotyping, a subset of 30 wheat accessions (Additional file 3) showing representative variation at the chosen marker loci, were selected for mechanical testing. These accessions were mechanically tested as described previously. Using a T-test (Genstat 15th edition) the trait data and genotype data obtained were assessed for any significant marker-trait segregation patterns.

## Results

### Variation for stem structural and material strength

The diversity panel of 100 wheat accessions was analysed for a range of traits indicative of stem structural and material strength. With the exception of second moment of area, significant variation was present for all traits included in the analysis (*P* < 0.05) (Additional file 1). The absolute strength traits Fmax and F/V showed respective trait ranges of 7.45–38.55 and 29.82–80.44 N/s. The wheat accession displaying highest stem absolute strength (for both Fmax and F/V) was Orlando. The lowest trait values were seen in Battalion and Escorial for F/V and Fmax respectively. For the material strength traits, MOR and MOE, respective trait ranges of 0.70–8.05 and 121.6–1490.3 Nmm^−2^ were recorded. Of the wheat accessions screened, Gatsby exhibited the lowest trait values for both MOE and MOR. Accessions displaying the highest material strength were Alba (for MOR) and Cordiale (for MOE). A wide range of variation was also observed for the various stem structural traits assessed. For example, mean stem hollow area ranged from 1.16 mm^2^ (for Capelle-Desprez) and 6.51 mm^2^ (for Starke2). For outer cortex thickness, trait means ranging between 0.24 mm (as seen for Hyperion) and 0.46 mm (as seen for Alba) were recorded. For plant height, despite a lack of segregation at the *Rht* loci, a trait range of 42.8–98.4 cm was recorded. The tallest accession included within the panel was Steadfast whereas the shortest stem measurements were recorded for Equinox.

A correlation analysis was performed to analyse the relationships between the absolute strength and the structural and morphological traits to assess which may be good breeding targets (Table 1). Several highly significant (*P* ≤ 0.001) relationships were detected between the absolute strength measures (Fmax and F/V) and the structural traits, however, despite such high statistical significance, in the majority of cases, the amount of variation in stem absolute strength explained by stem structure was found to be modest. Stem parenchyma area (R^2^ = 0.27 and 0.17 for Fmax and F/V respectively) and outer cortex thickness (R^2^ = 0.19 and 0.13 for Fmax and F/V respectively) show the closest positive relationships with absolute strength. These traits may therefore be the most promising targets for the improvement of stem structural strength in wheat. In contrast to the modest contributions made by stem geometry, a much closer correlation is seen between the absolute strength measures and stem weight (R^2^ = 0.42 and 0.47 for Fmax and F/V respectively). These correlations may represent a combined effect of several different stem structural components (each contributing to weight) or may more specifically relate to the density of the materials that make up the plant stem. Plant height also correlates positively with stem absolute strength (R^2^ = 0.21 and 0.25 for Fmax and F/V respectively).

**Table 1:** Pearson’s correlation coefficient (tested against zero) for traits measured across wheat panel

Fmax (N/s)										
F/V (N/s)	***0.85									
Stem width (mm)	0.01	0								
Stem hollow area (mm^2^)	***0.16	***0.12	***0.27							
Second moment of area (N/mm^4^)	**0.07	*0.06	***0.33	***0.16						
Parenchyma area (mm^2^)	***0.27	***0.17	***0.11	***0.11	**0.09					
Outer cortex thickness (mm)	***0.19	***0.13	0.032	0	0.02	0				
Length of 2nd internode (cm)	0.014	0.037	0	**0.09	*0.06	*0.06	0			
Height minus ear (cm)	***0.21	***0.25	0.011	0.01	**0.09	0	**0.08	***0.38		
Threshed stem weight (g)	***0.49	***0.51	***0.13	0.01	***0.22	***0.15	**0.09	**0.1	***0.55	
	Fmax (N/s)	F/V (N/s)	Stem width (mm)	Stem hollow area (mm^2^)	Second moment of area (N/mm^4^)	Parenchyma area (mm^2^)	Outer cortex thickness (mm)	Length of 2nd internode (cm)	Height minus ear (cm)	Threshed stem weight (g)

*** indicates significance at *P* ≤0.001 and ** indicates significance at *P* ≤0.01 and * indicates significance at *P* ≤ 0.05

The lack of strong correlations observed between stem structure and absolute strength may suggest that stem material properties are of high value for the improvement of stem mechanical strength in wheat. Consistent with this, the relationship between the field-based measure of stem lodging risk (utilising the pulley system illustrated in Fig. 1c) and the absolute and material strength traits, showed a stronger correlation for the material strength trait Modulus of Rupture (MOR; R^2^ of 0.41, *P* < 0.001) in comparison to absolute strength traits such as Fmax (R^2^ of 0.27, *P* < 0.001) (Additional file 4).

### The development of functional genotypes for Associative Transcriptomics

Illumina mRNAseq data were produced from leaf RNA from the diversity panel of 100 wheat accessions. These sequences were mapped to the ordered transcriptome reference reported recently (Harper et al., [9]), with an average number of input reads across the full panel of 29.5 million, providing an average read coverage of 5.87. The panel was scored for SNPs and transcripts were quantified as RPKM. In total, 42,066 SNPs were scored, of which 12,456 were present at MAF > 0.05, so were considered suitable for use in AT. Abundance was measured as >0.4 RPKM across the population for 94,060 transcripts, which were considered suitable for use in AT. Full association plots for the following traits can be found in Additional file 5: Figures S1–S9.

### Associative Transcriptomics for plant height

**Fig. 2:**
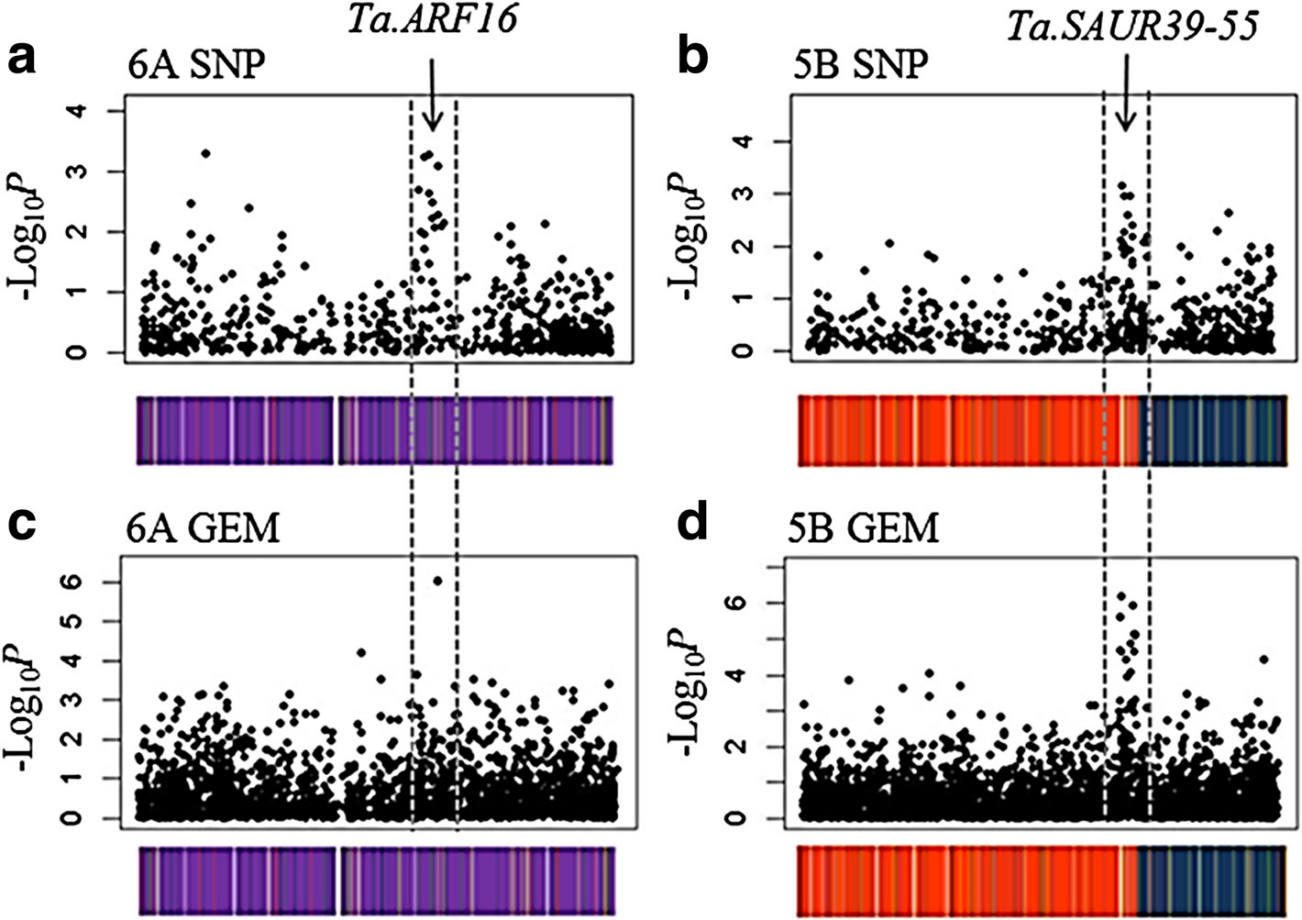
SNP and GEM marker associations detected for plant height. Marker associations are illustrated, for both sequence-based (SNP) and gene expression-based (GEM) markers, with significance of association (as –log10*P* values) plotted against position within specific chromosomes. The inferred order of unigenes is illustrated below the scans with colour coding by sequence similarity to chromosomes of *B. distachyon* (*blue* = Bd1; *yellow* = Bd2; *purple* = Bd3; *red* = Bd4 and *green* = Bd5). Two associating loci for height are shown, one on chromosome 6A (**a**, **c**) and one on chromosome 5B (**b**, **d**). Both loci show associating SNP and GEM marker variation. The positions of candidate genes are indicated by arrows

In order to identify loci controlling plant height, AT was conducted using the functional genotypes scored and the plant height trait data obtained. Additional file 6 summarises the results obtained. Two major association peaks were identified: one on chromosome 6A and the other on 5B, each exhibiting SNP and GEM associations (Fig. 2). To identify candidates for the causative genes for control of the trait underlying the association peaks, the sequence similarities of unigenes to gene models in Brachypodium, Sorghum, rice and Arabidopsis were used as a guide to gene function. This revealed that the gene corresponding to the highest significance GEM on 6A is an orthologue of a rice Auxin Response Factor (*OsARF16*, Os02g41800; Panel a). The peak found on chromosome 5B coincided with a cluster of *SMALL AUXIN UP RNA* (SAUR) genes, with high significance GEMs occurring in three of the unigenes with BLAST identity to SAUR genes (Panel b). Although these loci have not been implicated previously in the control of plant height in wheat, the genes identified are excellent candidates for controlling this trait: ARFs are transcription factors that bind specifically to auxin response elements (*AuxRE*s) found in the promoters of early auxin response genes such as the large family of SAUR genes, and mediate their response to auxin [20]. In wheat, we found that the GEM for the ARF on 6A had a positive correlation with stem height. These results suggest that this Auxin Response Factor may have a developmental role in wheat. Although the actual function of the SAURs is not known, it has been reported that some have an important role in control of cell expansion and patterning [21]. On closer inspection of their sequence similarities, the SAUR genes in the region of 5B are putative orthologues of some of the members of a cluster of 17 SAURs found on rice chromosome 9 (*OsSAUR39-55*) and an orthologous cluster can also be found on Arabidopsis chromosome 1 (*AtSAUR61-68*) [22]. In rice, *OsSAUR39* has been found to negatively regulate auxin synthesis and transport, leading to reduced growth phenotypes when over-expressed [23]. Our observation that all of the highly associated SAURs in this cluster exhibited gene expression that was negatively correlated with height is concordant with this.

### Associative Transcriptomics for modulus of rupture

**Fig. 3:**
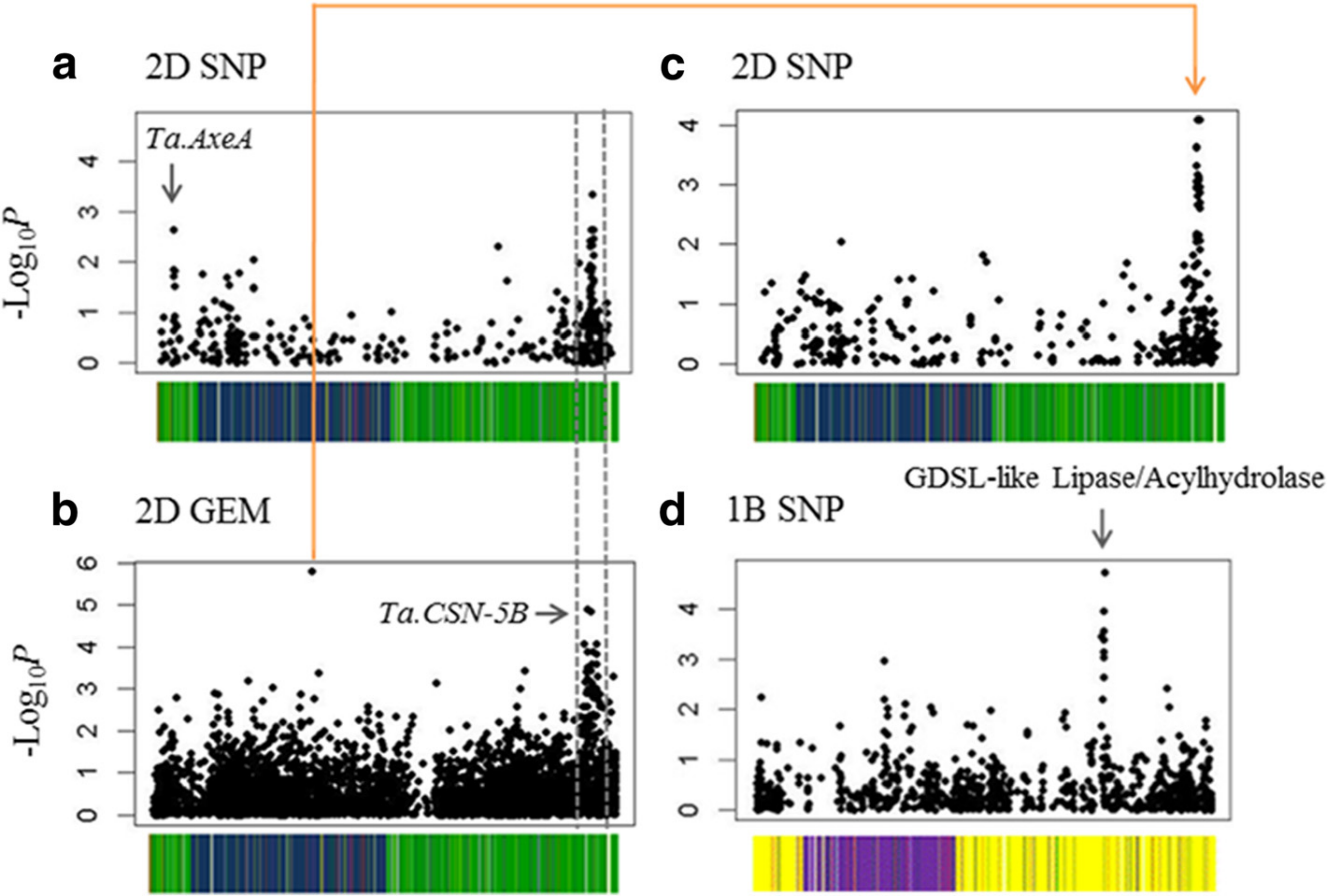
Variation at both the sequence (SNP) and gene expression (GEM) level show high association with MOR. Two SNP association peaks for MOR were seen on chromosome 2D (**a**). The peak to the right of panel a was also identified in the GEM analysis (**b**). Several single GEM associations were also detected for MOR (see single GEM at the foot of the orange line in panel **b** as an example). Mapping transcript abundance (as RPKM) as a trait against the SNP data revealed the same 2D SNP peak for several single GEMs (see panel **c** for an example). A further SNP association for MOR was detected on chromosome 1B **(d)**. The positions of candidate genes are indicated by *arrows*. -Log10*P* values are plotted in wheat pseudomolecule order. Unigene order is colour-coded according to sequence similarity to *B. distachyon* chromosomes (*blue* = Bd1; *yellow* = Bd2; *purple* = Bd3; *red* = Bd4 and *green* = Bd5). Position of candidate genes are indicated by arrows

AT for MOR identified three SNP association peaks. On chromosome 2D, two association peaks were found. The first of these (marked with an arrow in Fig. 3a) was found to be in close proximity to a gene orthologous to a rice acetyl xylan esterase (*AxeA;* Os04g01980). *AxeA*, is thought to have hydrolase activity, specifically acting on ester bonds in the deacetylation of xylans in the plant cell wall [24]. The second association peak found on chromosome 2D for MOR exhibited both SNP and GEM associations (shown within the grey dotted lines on Fig. 3a and b). Several genes in this region show a consistent, positive, relationship of their expression with variation in MOR, which may be indicative of a large-scale rearrangement such as a deletion.

A final SNP association peak was seen on chromosome 1B (Fig. 3d). On closer inspection, it was revealed that the locus with the most highly associated marker has high sequence similarity to an Arabidopsis GDSL-like Lipase/Acylhydrolase superfamily gene (At1g54790). GDSL-like lipases are thought to be involved in the hydrolysis of ester bonds in cell wall xylans and have been found to have xylan acetylase activity [25]. This is a very similar function to that previously described for the candidate detected on chromosome 2D. Previous work in Arabidopsis has shown that xylan acetylation is an important contributor to stem strength. For example, the *eskimo-1* mutant, which displays reduced xylan acetylation, exhibits reduced cell wall thickening and significantly weaker stems in comparison to wild-type plants [26].

In addition to the GEM association peak seen on chromosome 2D, several individual GEMs were also found to show significant association with material strength. An example of this can be seen in Fig. 3b (GEM marked at the foot of orange line). Transcript abundance for this GEM correlates positively with MOR. This marker corresponds to an orthologue of Arabidopsis *SERINE CARBOXYPEPTIDASE-LIKE 49* (At3g10410). The Tobacco orthologue of this gene, *NtSCP1*, is known to be important for cell elongation and it has been proposed that this gene may target proteins involved in cell wall remodelling [27], making this a very plausible candidate gene for stem material strength. Another example was found on chromosome 7B with a GEM corresponding to an orthologue of Arabidopsis *QUASIMODO 1* (At3g25140). Mutants defective in this gene exhibit a number of defects including reduced homogalacturonan (a cell wall pectin) content in the cell wall and reduced cell adhesion [28]. Previous studies have shown that variation in pectin can have a dramatic effect of stem mechanical strength in plants [29]. As a final example, on chromosome 6B, a marker located within a gene orthologous to that which, in rice, has been described as a translation initiation factor, *EIF-2B* epsilon subunit (Os02g56740), shows a high association with MOR. In rice, this gene is thought to have a role in the recruitment of mRNAs and the machinery required for translation. A related protein however, *EIF-5A*, has been found to be involved in a signalling pathway contributing to cell wall integrity and formation [30]. It is therefore possible that *EIF-2B* also has a similar, additional function.

To further analyse the individual GEM associations detected, their respective transcript abundances (measured as RPKM) were mapped as traits against the SNP data of the wheat accessions. Interestingly, for each of the described GEMs, a strong SNP peak was detected on chromosome 2D, the same region previously described for MOR in both the SNP and GEM analyses. An example of this can be seen in Fig. 3c for the previously mentioned single GEM detected on chromosome 2D (Fig. 3b). Figure 3c shows a clear SNP association on chromosome 2D following the mapping of the transcript abundance values for this GEM as a trait against the SNP data. All additional GEMs found to show this relationship with the 2D locus can be seen marked with a red asterisk in Additional file 6. This finding could be indicative of an interaction between those genes detected as single marker associations and one or more genes located within the 2D region. Due to the many genes showing associations in the 2D region detected in the GEM analysis for MOR, it is difficult to propose a candidate gene. However, one gene, which corresponds to one of the most highly associating GEM markers within this peak, may be considered a very plausible target. This gene corresponds to an orthologue in rice described as a *COP9 SIGNALOSOME SUBUNIT 5B* (*CSN5B*) (Os04g56070). The COP9 signalosome is a multi-protein complex which is known to be involved in protein degradation and has, in plants, been implicated in a number of developmental processes including photomorphogenesis (light-mediated growth), cell cycle progression and gene expression [31]. Interestingly, in Fungi, the COP9 signalosome has been implicated in cell wall remodelling. Work conducted by Nahlik et al., found that in the absence of a functional COP9 complex, *Aspergillus nidulans* exhibits altered expression of genes involved in cell wall remodelling [32]. Furthermore, one of the single GEM associations detected for material strength, corresponds to a eukaryotic translation initiation factor (*EIF-2B*) gene. Previous studies have shown evidence of interactions between EIF-related genes and the COP9 complex [33]. Given this, it is plausible that the genes associated with material strength are interacting with the COP9 (or more specifically, *CSN5B*) complex by means of a pathway analogous to that seen in *Aspergillus nidilans*, contributing to cell wall remodelling.

### Marker validation

To test the power of Associative Transcriptomics for the identification of predictive markers, a marker validation study was carried out using a panel of 96 additional wheat accessions and focusing on three SNP associations previously described for MOR. This analysis involved the screening of a completely independent panel of wheat accessions (taken from the WAGTAIL panel) for variation at the three marker loci. These accessions were then phenotyped using the three-point bend test as before and any marker-trait segregation patterns assessed statistically.

Although this analysis would ideally focus on segregating variation of the most significant SNP within the association peak, the development of genome-specific marker assays for two of the targeted loci (B_comp6657_c0_seq1:3733 and For D_comp970_c0_seq1:1030) proved problematic (due to mixed traces in sequencing reads). However, genome-specific marker assays were successfully developed for alternative, highly associating SNPs within the corresponding peaks. Additional file 7: Table S1 provides an overview of the marker assays used for successful amplification of the targeted loci. Although variation was seen for two of the targeted marker loci, the WAGTAIL panel was monomorphic for D_comp1058_c0_seq1:1573, so it was not used.

Based on the marker variation uncovered from the remaining two marker assays, 30 accessions were chosen for mechanical testing. These accessions were chosen based on genotype alone to ensure non-biased trait prediction and to ensure that all possible marker allele combinations were represented in downstream analyses. Following mechanical testing, a student T-test was used to assess whether, on average, a higher trait value is observed in accessions carrying the increasing alleles of the markers uncovered through AT, thus proving that the markers identified have trait predictive capability. Additional file 5: Figure S10 summarises the results for each marker locus. As predicted, significantly increased trait values are seen in segregation with increasing alleles at both loci (with segregation patterns being assigned *P* values of ≤0.01 and ≤0.001 for D_comp19374_c0_seq1:702 and B_comp2391_c0_seq1:284 respectively), proving that these markers have robust trait prediction capability. It is also promising to note, that the WAGTAIL accessions showing particularly high mean MOR (between 25.9 and 34.9 N mm^−2^), are among those carrying increasing alleles at both marker loci (Additional file 5: Figure S11).

## Discussion

Despite great efforts, lodging continues to be one of the key factors threatening wheat yield worldwide. The selection of elite accessions with alternative semi-dwarfing alleles or high stem mechanical strength may be a powerful approach to reducing this problem.

As previously mentioned, the selection of dwarfing alleles is a commonly employed method for lodging control in wheat. The lack of segregation of these loci (*Rht-B1 and Rht-D1*) in our training panel has enabled the identification of additional candidate genes that may contribute to controlling height in this species, implicating auxin-related genes as key regulators. Importantly, the loci implicated in plant height control are completely independent to those seen for stem strength and may therefore be used to further maximise lodging resistance in future elite wheat accessions, or to develop taller lodging resistant accessions. Such accessions would also improve the achievable profit margin by increasing the amount of straw that can be harvested for use as animal bedding or biorefining feedstocks.

In recent years we have seen increased interest in the possible exploitation of agricultural residues (such as waste straw of the wheat crop) as a feedstock for lignocellulosic ethanol production. However, at present, high costs related to the breakdown of lignocellulosic biomass is hindering this fuel source becoming a feasible future alternative. One way through which processibility may be improved, is through altering the composition of the lignocellulosic matrix. In this study, we have shown evidence for the importance of xylan acetylation in contributing to stem material strength in wheat, but xylan acetylation is also known to impede the enzymatic breakdown of lignocellulosic biomass and therefore reduced xylan acetylation is a desirable target for this industry [34]. The results presented here suggest that alterations in xylan acetylation may affect stem mechanical strength, so given this, it is essential that any effects of altering cell wall xylan acetylation on agronomic performance are assessed.

In addition to the potential role of xylan acetylation, this study has uncovered a possible role of the COP9 signalosome in contributing to stem mechanical strength in wheat. The detection of several interactions between single GEMs and the *CSN5* locus is very interesting. One of the associating GEMs showing a relationship with the 2D locus, corresponds to an orthologue of *EIF-2B*. Previous studies have shown evidence of interactions between the COP9 complex and EIF-related genes. This suggests that the utilisation of the GEM data for the assessment of gene-gene interactions through mapping is effective. Several of the GEMs found to interact with the 2D locus are expected to have a role in cell wall remodelling/biosynthesis. Previous work has shown that, in fungi, the COP9 complex has a role in cell wall remodelling, an important aspect of growth [32]. It is possible, based on the results presented here, that there is a pathway, analogous to that described in fungi, where the COP9 complex (or at the very least subunit *CSN5B*) regulates the expression of genes involved in cell wall remodelling, and that this is an important contributor to stem material strength in wheat. To our knowledge, this is the first instance of reporting such a role of the COP9 complex *in planta*.

## Conclusions

This work illustrates the power of Associative Transcriptomics for the exploration of even the most complex, environmentally sensitive traits. With careful selection of the genotypes included, we have shown that even a relatively small diversity set can, when coupled with high marker density and low linkage disequilibrium, provide the power required for the discovery of novel and agronomically valuable genetic variation. In this study, we successfully identified and validated markers for two loci that provide increased Modulus of Rupture, an important measure of the resistance of the plant material to breakage. We have also shown that this method has the potential to uncover novel targets for breeding of important morphological traits, such as plant height. Furthermore, the coupling of SNP variation with variation at the gene expression level has provided the power required to gain a deeper understanding of the biological context of variation underlying important agronomic traits. This not only adds to the wealth of knowledge that we strive to accumulate regarding gene function and plant adaptation, but also provides breeders with the information required to make more informed decisions regarding the potential consequences of incorporating the use of particular markers into future breeding programmes.

## Additional files

Additional file 1: Supplementary dataset 1. Mean trait data as inputted into association models is presented for all traits. Both accession names (as referred to in the text) and sequence identifiers (as names in sequence submissions) are given. (XLSX 22 kb) Additional file 2: Supplementary dataset 2. Q matrix as used for input in association models is provided. STRUCTURE was used to calculate the Q matrix using an admixture model with independent allele frequency. After 100,000 burn-in and 100,000 iterations of K = 1 to 5 were completed three times, the best value of K was determined by using the method of Evanno et al. [16]. The combined Q matrix for the three replicates for K = 2 are provided. (XLSX 11 kb) Additional file 3: Supplementary dataset 3. Trait data and alleles for SNP D_comp19374_c0_seq1:702 are provided for the Wagtail validation panel. (XLSX 15 kb) Additional file 4: Supplementary dataset 4. Data is provided for the field-based stem lodging risk score, and correlated with the stem strength measurements fmax and MOR. (XLSX 20 kb) Additional file 5: Figures S1–S11. Manhattan plots for all traits analysed by AT. (PDF 3653 kb) Additional file 6: Supplementary dataset 5. Summary of the best SNP and GEM association results. SNP results include p values, effect sizes, and increasing alleles. GEM results include p values, R2 values, and correlation trend (positive or negative). All results are provided with marker chromosome number and most likely candidate genes. (XLSX 22 kb) Additional file 7: Table S1. Marker variation screened across WAGTAIL accessions for marker validation. Primer sequences shown proved effective in screening targeted variation. (DOCX 13 kb)
