# Supplementary material for: Elucidation of the genetic basis of variation for stem strength characteristics in bread wheat by Associative Transcriptomics
Source: BMC Genomics. 2016 Jul 16;17:500. doi: 10.1186/s12864-016-2775-2 (PMC4947262; doi:10.1186/s12864-016-2775-2)
Supplement: Additional file 5: — Figures S1–S11. Manhattan plots for all traits analysed by AT. (PDF 3653 kb) [file 12864_2016_2775_MOESM5_ESM.pdf]

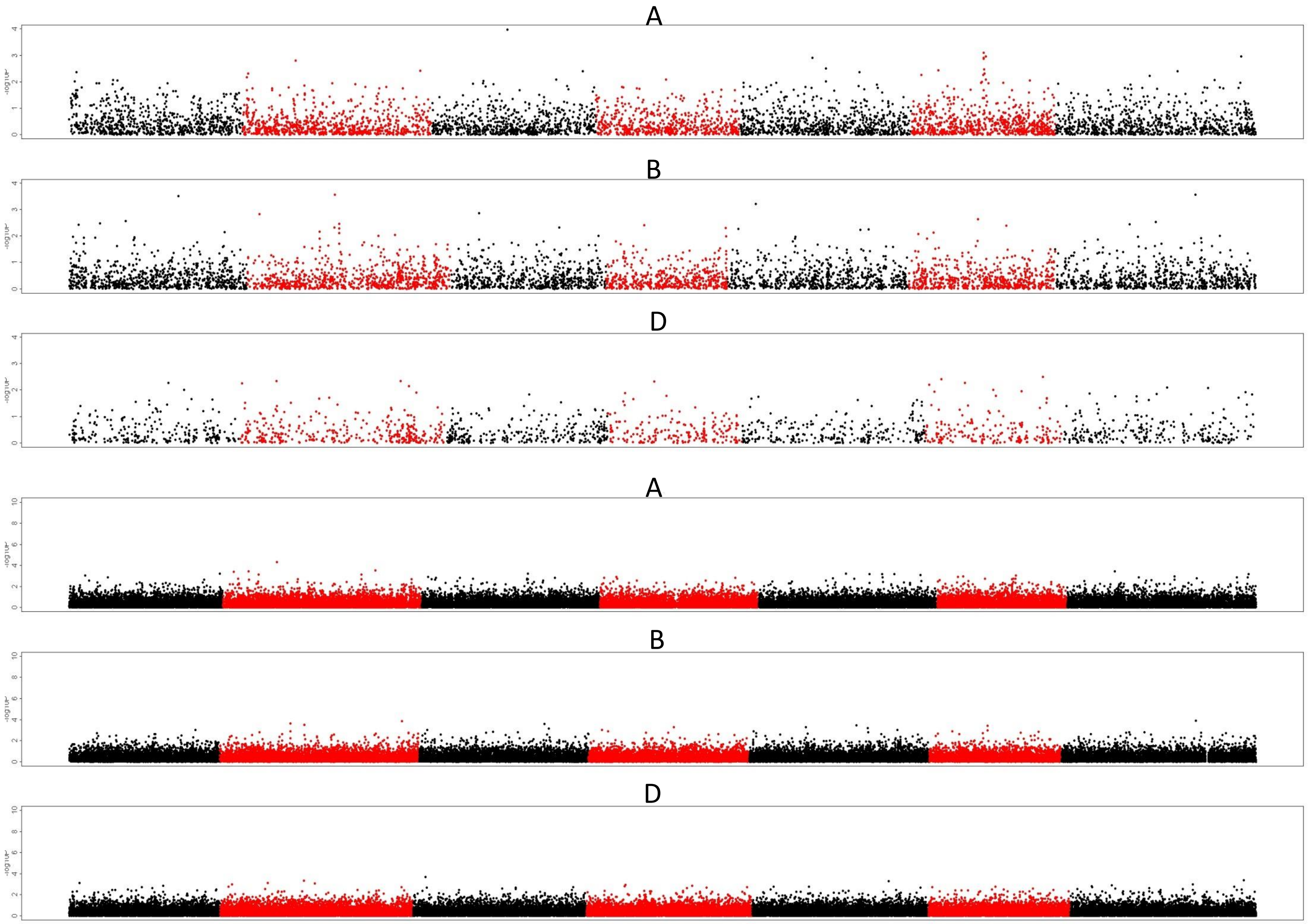

Supplementary Figure 1. SNP (top three panels) and GEM (bottom three panels) Manhattan plots for stem outer cortex thickness. The three rows represent each of the three genomes (labelled A, B and D), and individual chromosomes are indicated by alternating colours. The markers are arranged in pseudomolecule order.

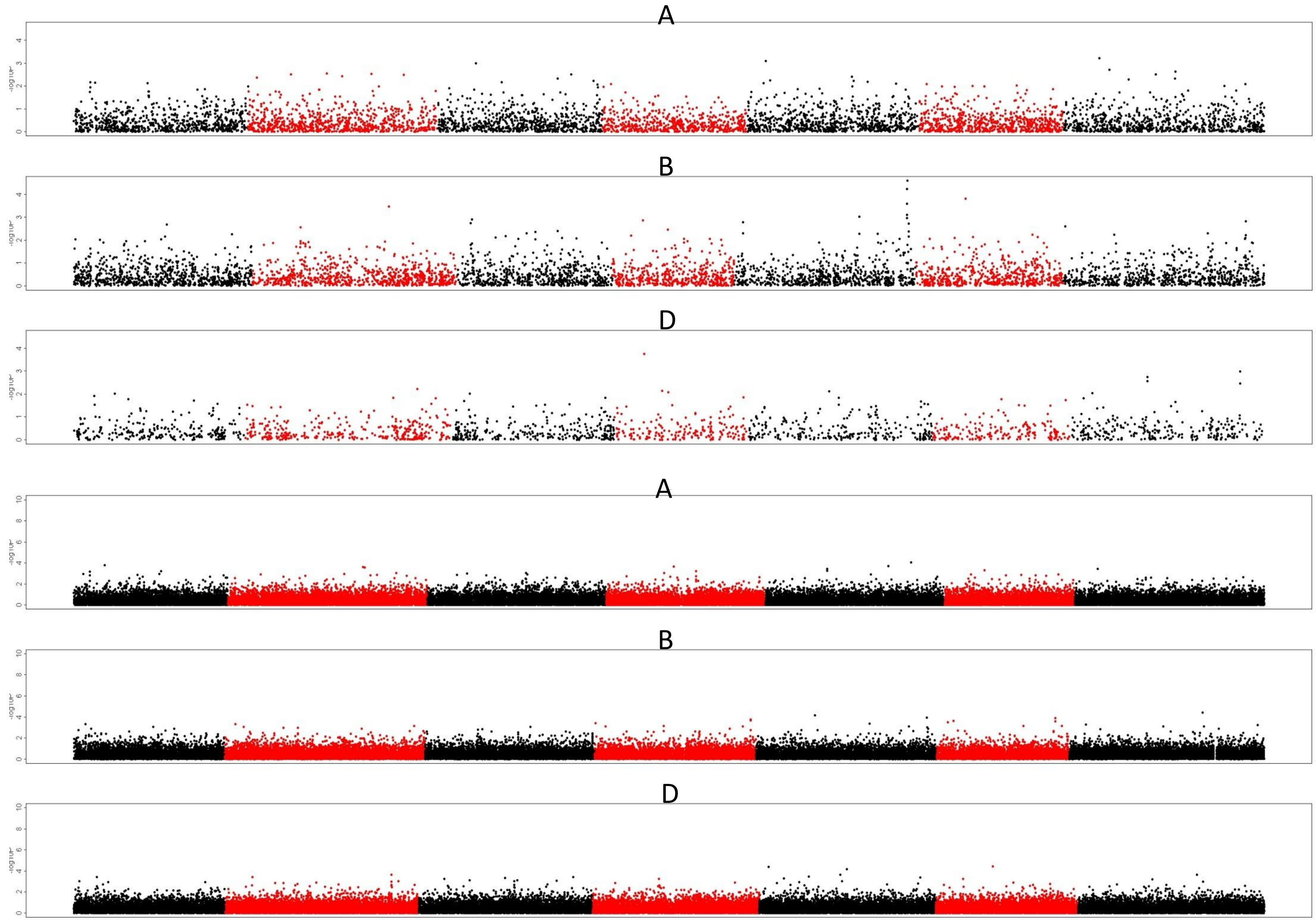

Supplementary Figure 2. SNP (top three panels) and GEM (bottom three panels) Manhattan plots for stem parenchyma area. The three rows represent each of the three genomes (labelled A, B and D), and individual chromosomes are indicated by alternating colours. The markers are arranged in pseudomolecule order.

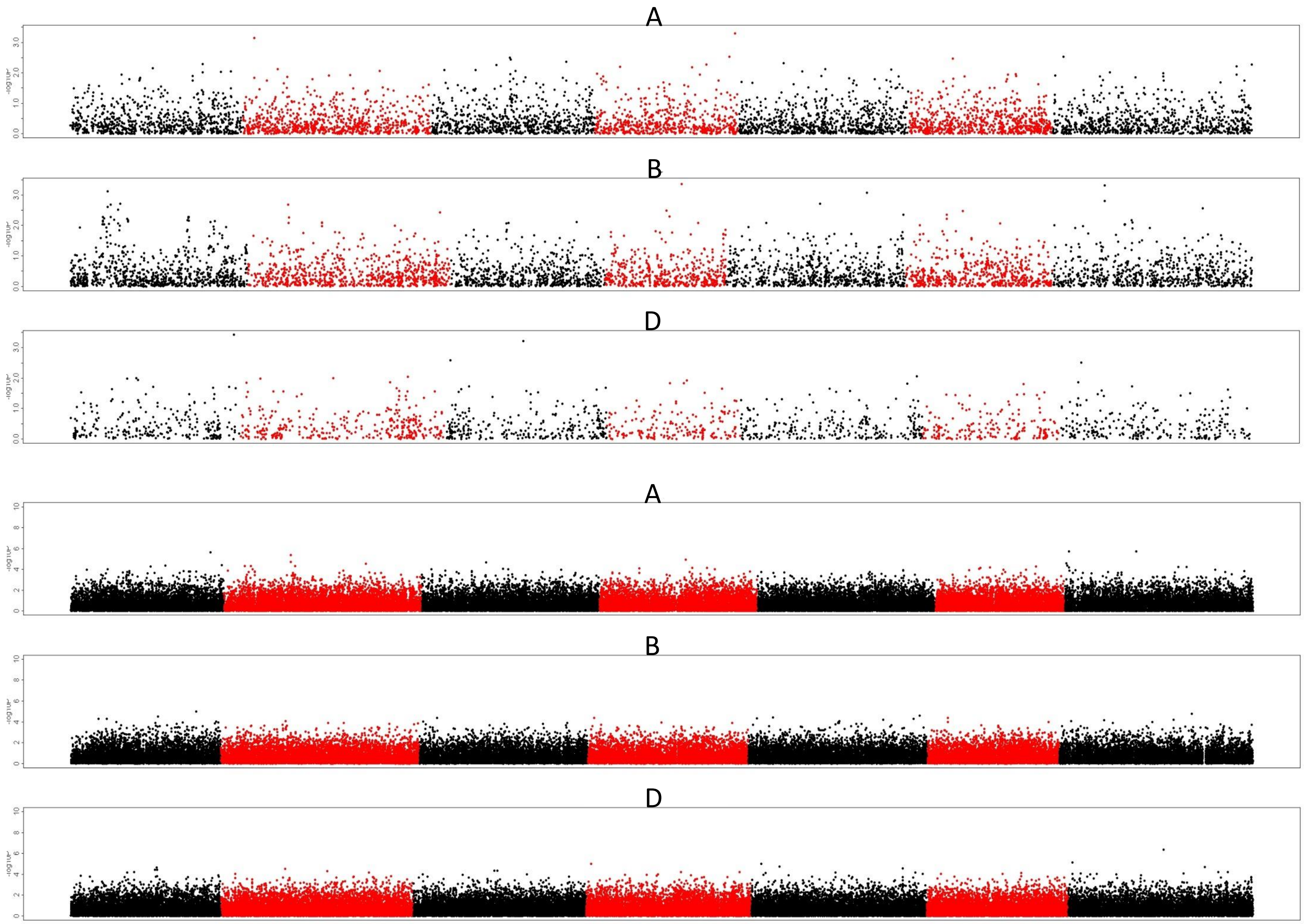

Supplementary Figure 3. SNP (top three panels) and GEM (bottom three panels) Manhattan plots for for stem hollow area. The three rows represent each of the three genomes (labelled A, B and D), and individual chromosomes are indicated by alternating colours. The markers are arranged in pseudomolecule order.

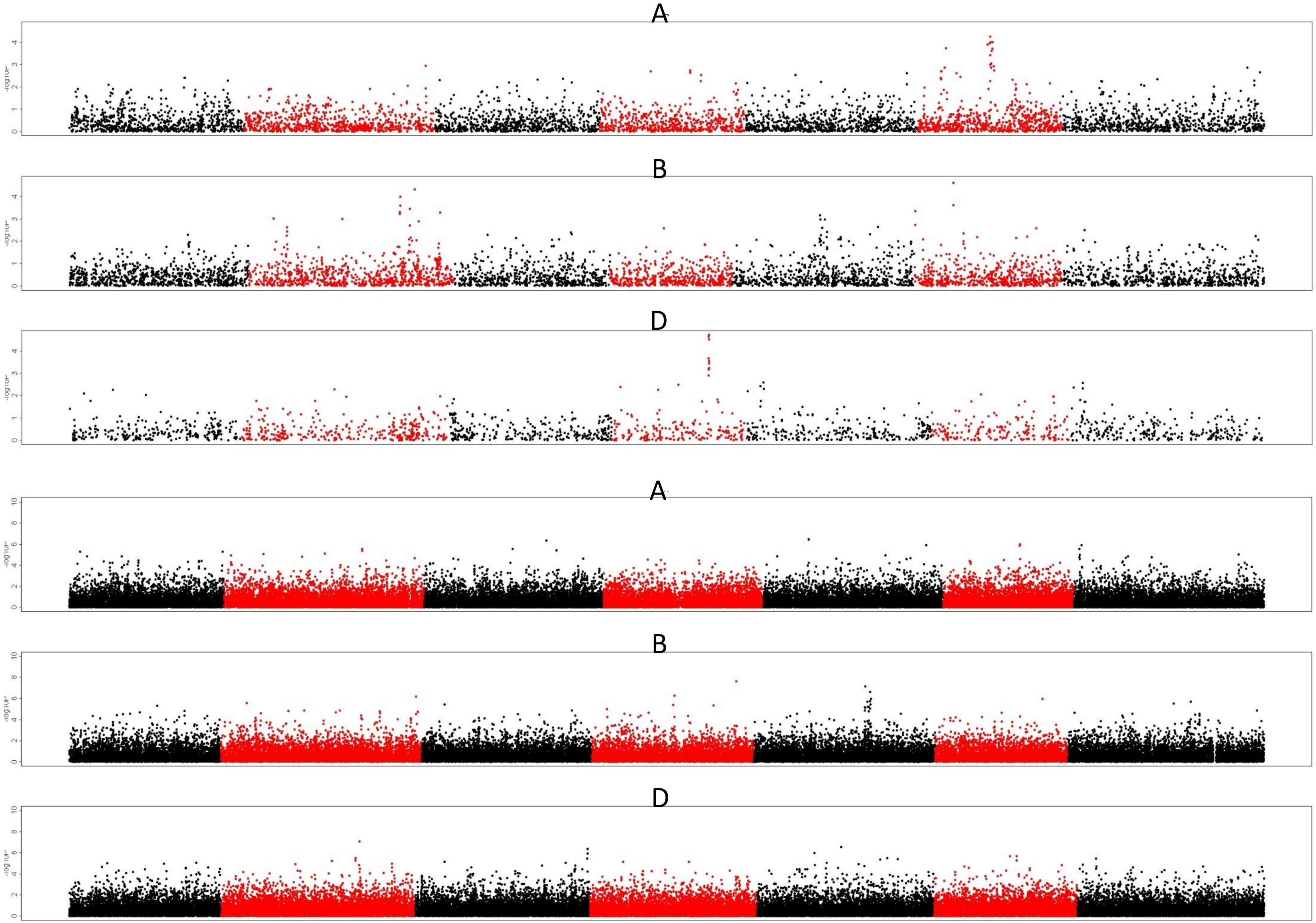

Supplementary Figure 4. SNP (top three panels) and GEM (bottom three panels) Manhattan plots for plant height. The three rows represent each of the three genomes (labelled A, B and D), and individual chromosomes are indicated by alternating colours. The markers are arranged in pseudomolecule order.

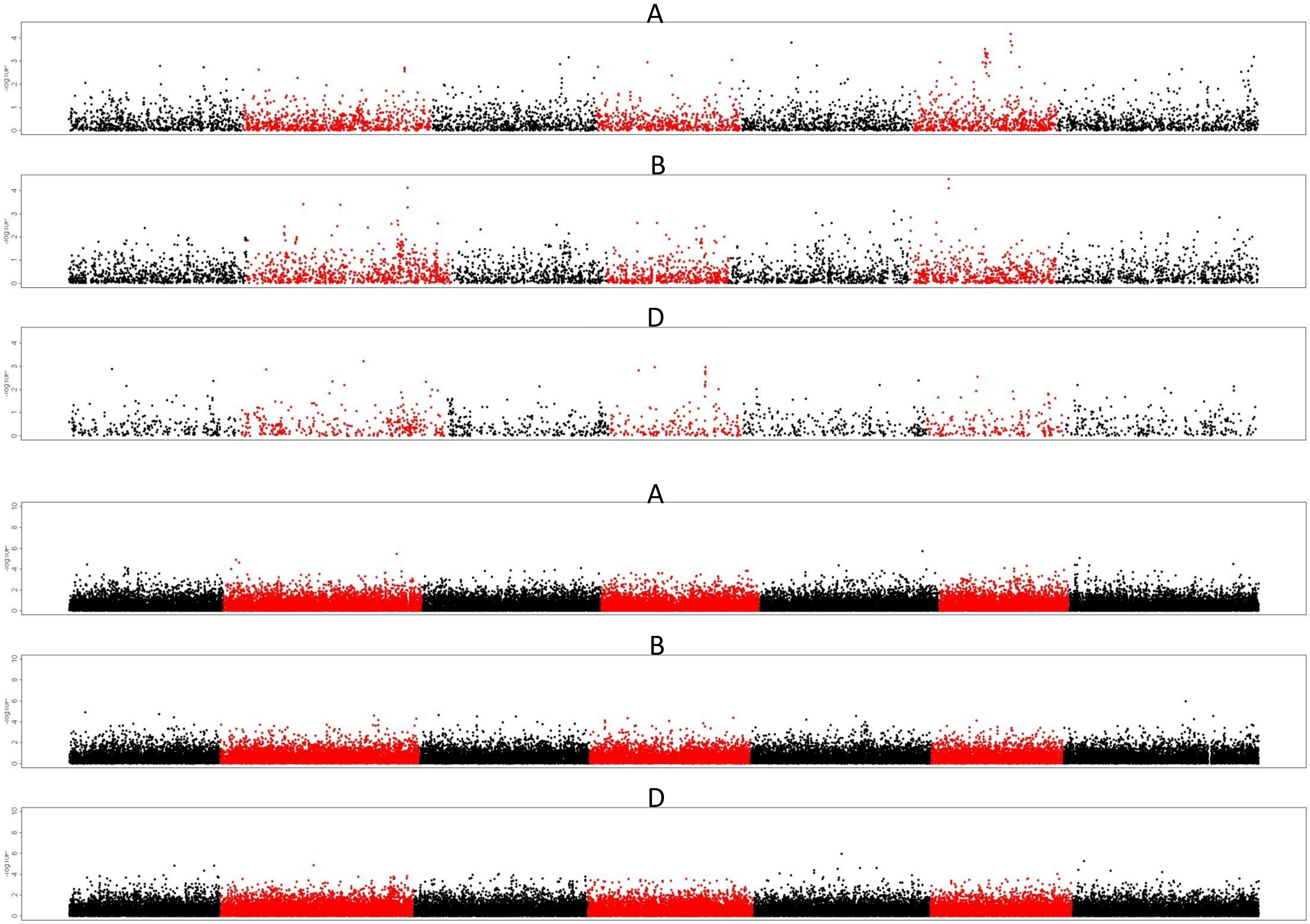

Supplementary Figure 5. SNP (top three panels) and GEM (bottom three panels) Manhattan plots for threshed stem weight. The three rows represent each of the three genomes (labelled A, B and D), and individual chromosomes are indicated by alternating colours. The markers are arranged in pseudomolecule order.

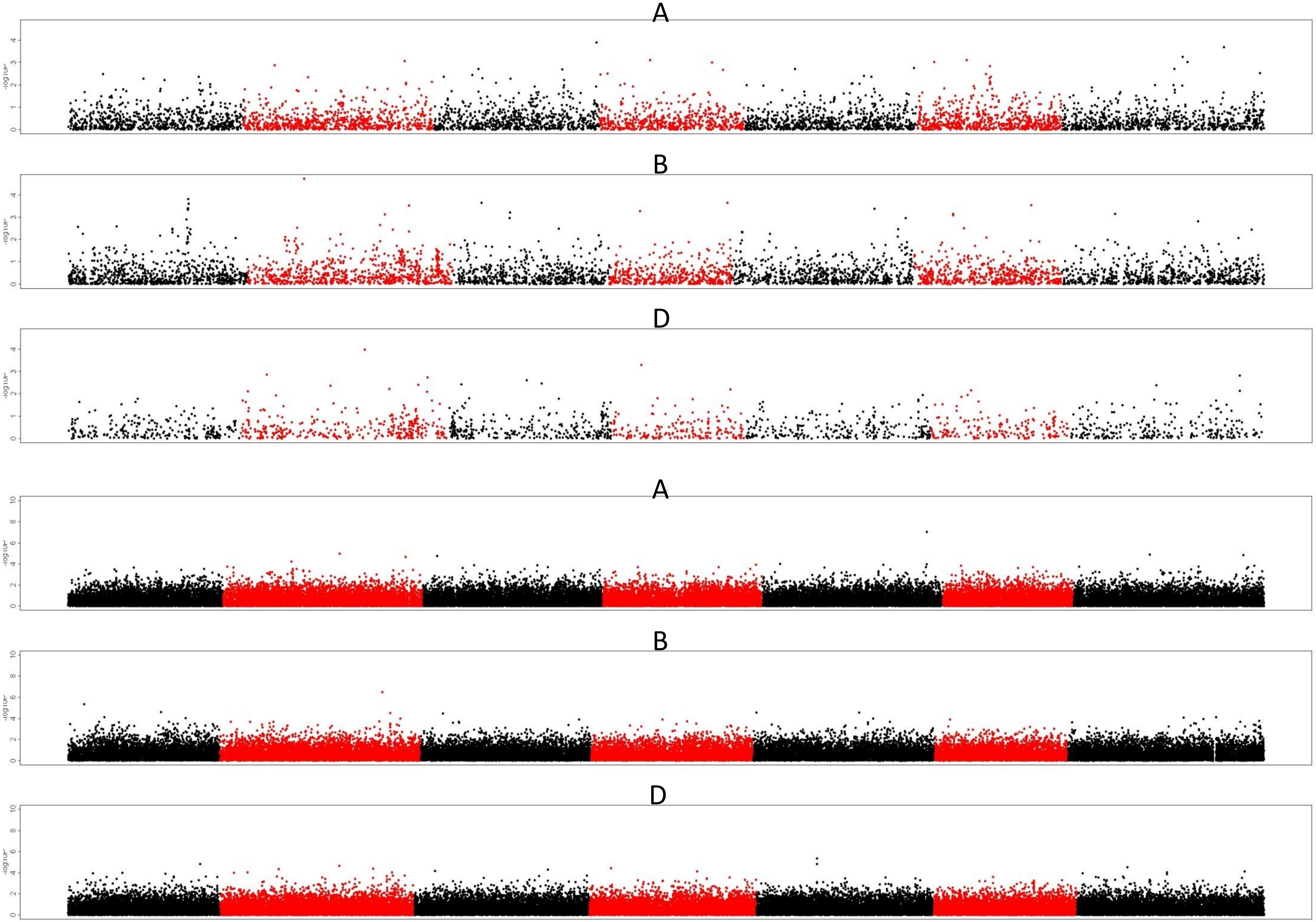

Supplementary Figure 6. SNP (top three panels) and GEM (bottom three panels) Manhattan plots for fmax. The three rows represent each of the three genomes (labelled A, B and D), and individual chromosomes are indicated by alternating colours. The markers are arranged in pseudomolecule order.

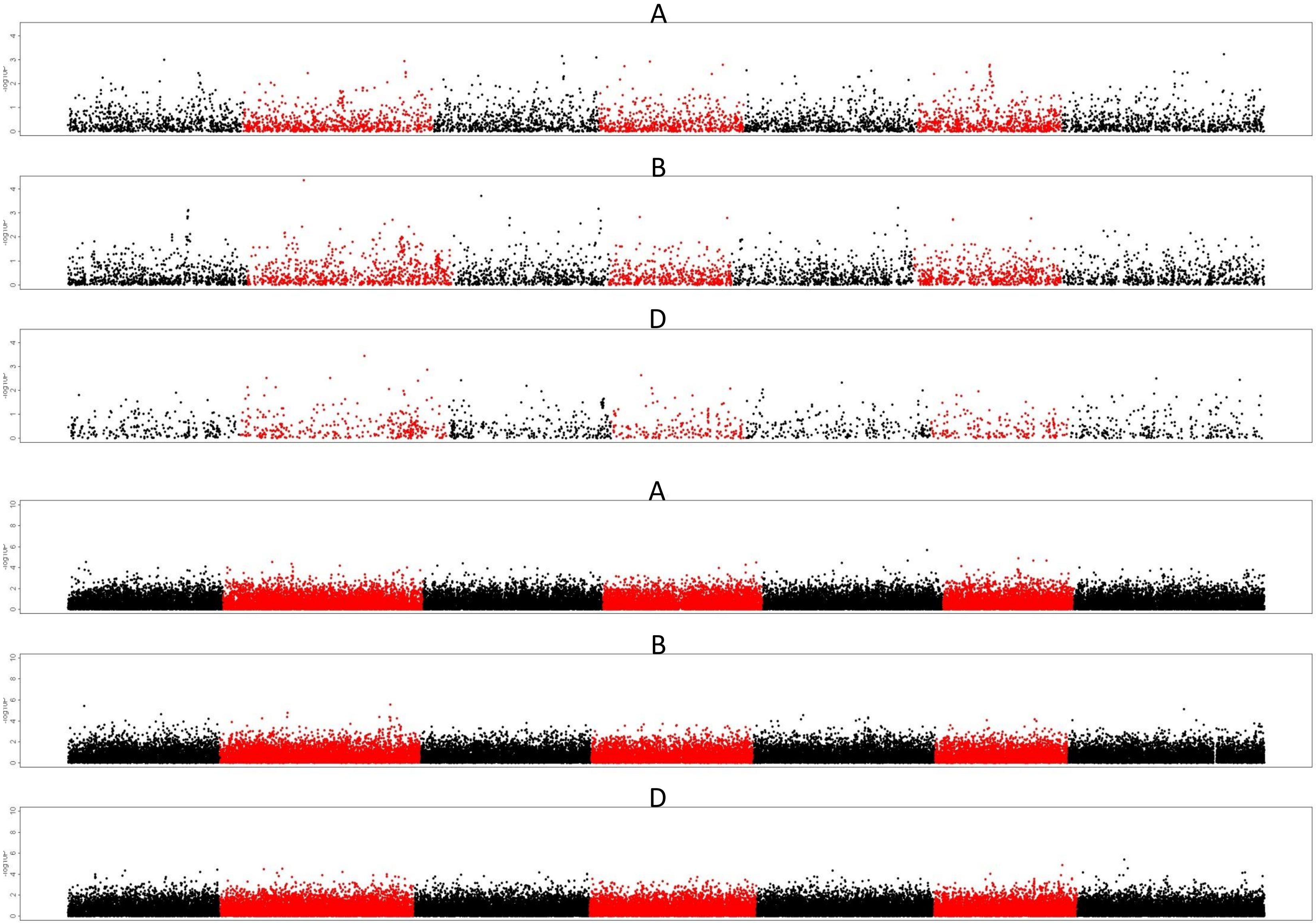

Supplementary Figure 7. SNP (top three panels) and GEM (bottom three panels) Manhattan plots for f/v. The three rows represent each of the three genomes (labelled A, B and D), and individual chromosomes are indicated by alternating colours. The markers are arranged in pseudomolecule order.

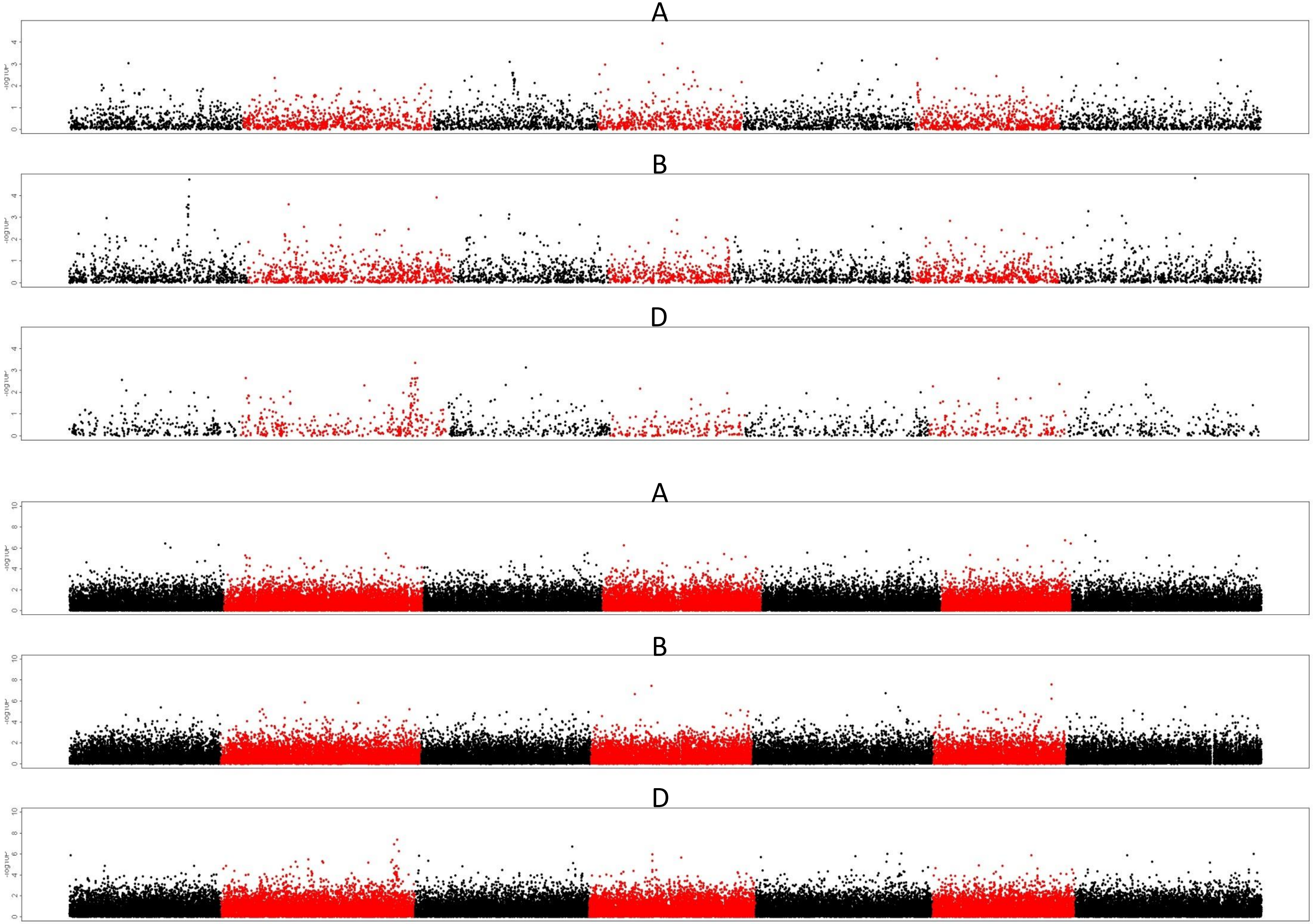

Supplementary Figure 8. SNP (top three panels) and GEM (bottom three panels) Manhattan plots for MOR. The three rows represent each of the three genomes (labelled A, B and D), and individual chromosomes are indicated by alternating colours. The markers are arranged in pseudomolecule order.

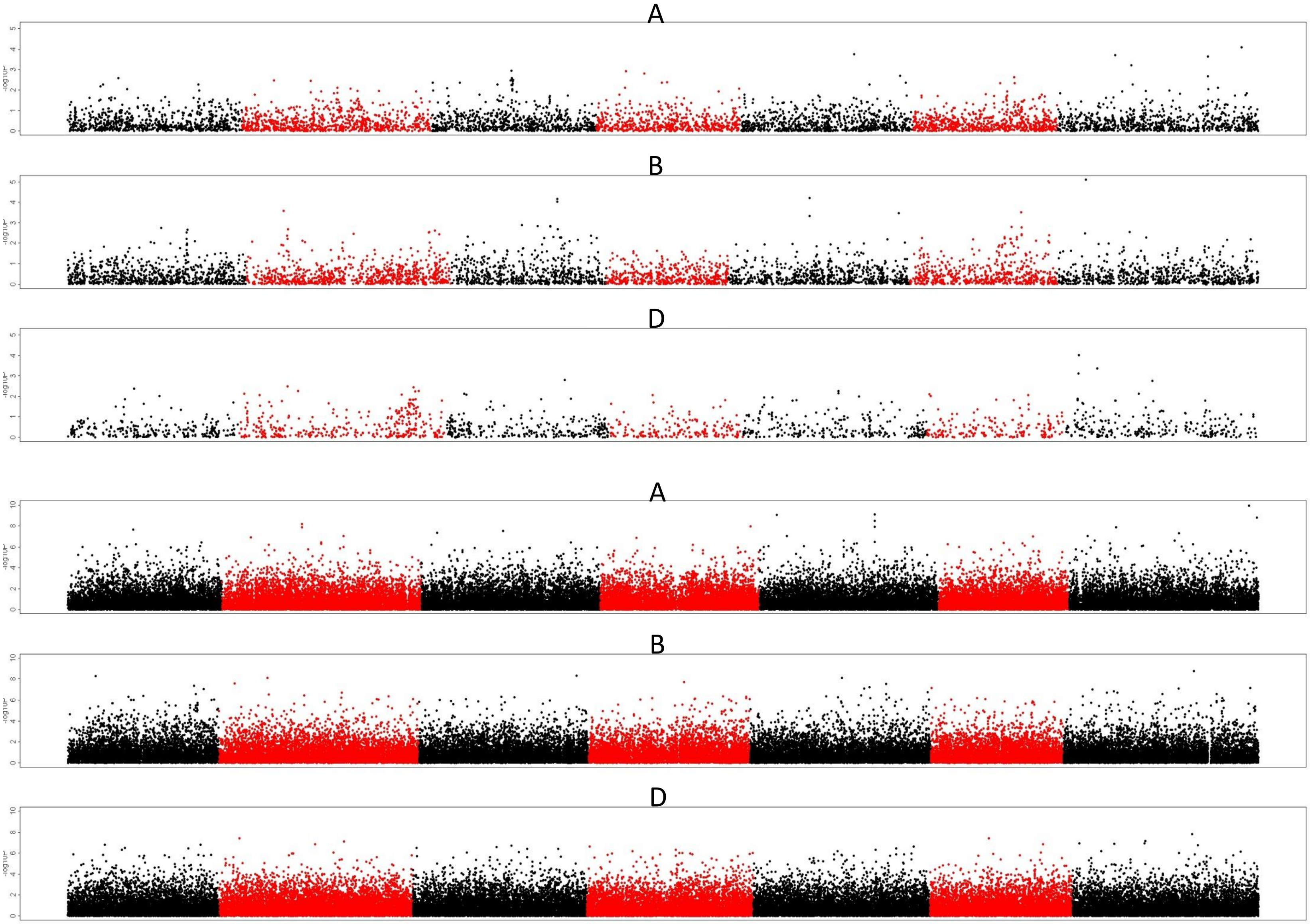

Supplementary Figure 9. SNP (top three panels) and GEM (bottom three panels) Manhattan plots for MOE. The three rows represent each of the three genomes (labelled A, B and D), and individual chromosomes are indicated by alternating colours. The markers are arranged in pseudomolecule order.

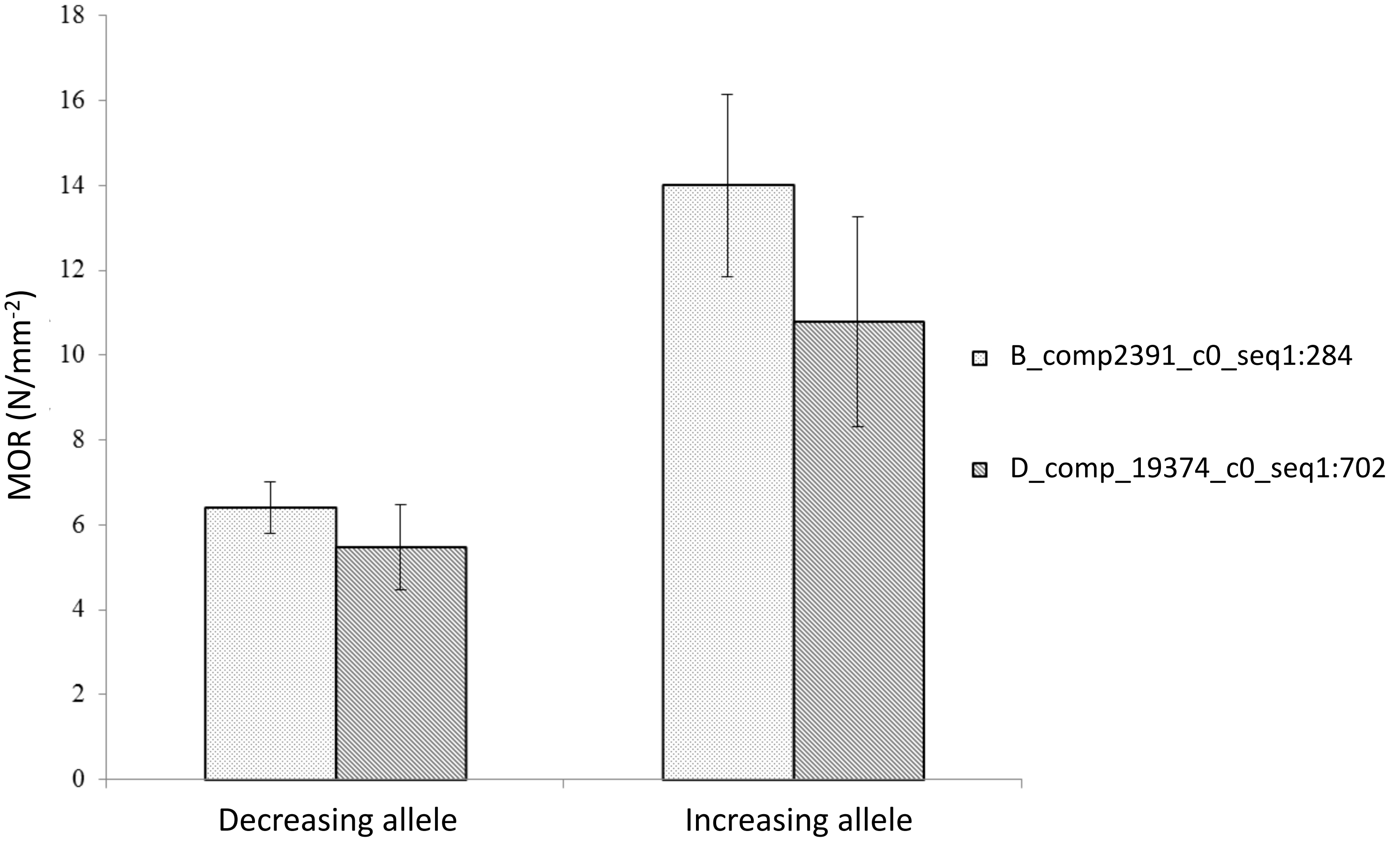

Supplementary Figure 10. Mean MOR values observed in WAGTAIL validation panel. Results are presented for the different alleles of two genes originally identified using Associative Transcriptomics. Error bars represent SEM.

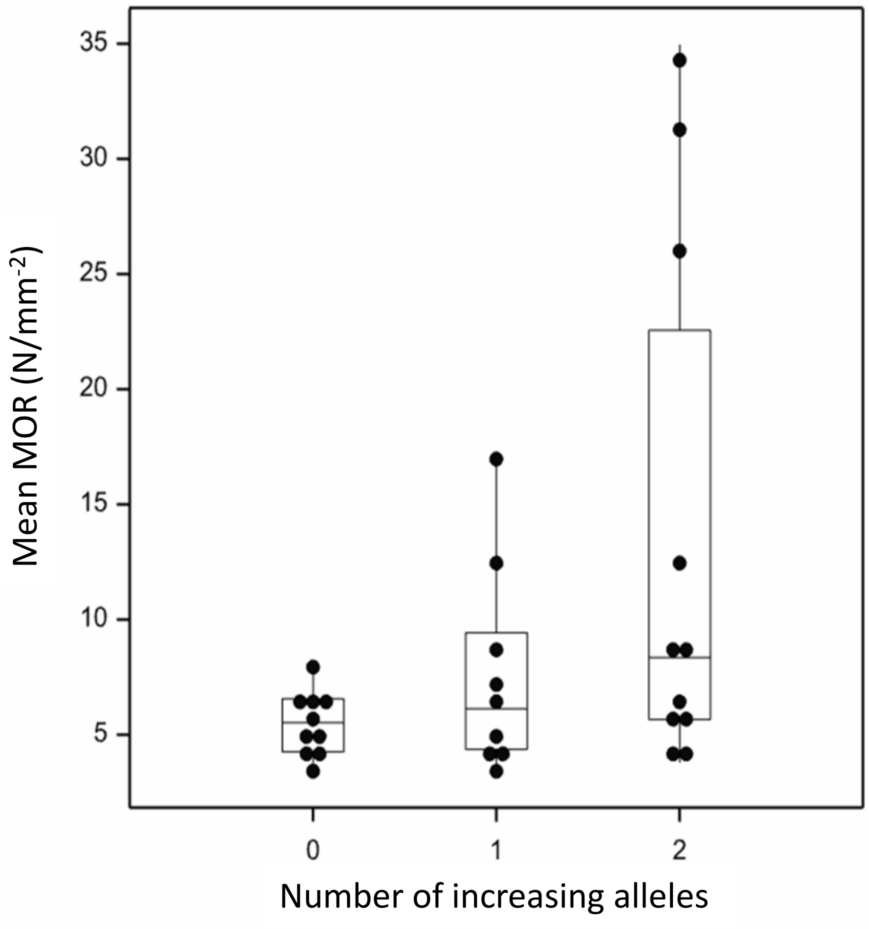

Supplementary Figure 11. Dot histogram boxplot showing mean MOR values observed in WAGTAIL validation panel for individuals with zero, one or two increasing alleles.
