# Supplementary material for: Elucidation of the genetic basis of variation for stem strength characteristics in bread wheat by Associative Transcriptomics
Source: BMC Genomics. 2016 Jul 16;17:500. doi: 10.1186/s12864-016-2775-2 (PMC4947262; doi:10.1186/s12864-016-2775-2)
Supplement: Additional file 7: Table S1. — Marker variation screened across WAGTAIL accessions for marker validation. Primer sequences shown proved effective in screening targeted variation. (DOCX 13 kb) [file 12864_2016_2775_MOESM7_ESM.docx]

| **Marker ID** | **Increasing allele** | **Decreasing allele** | **Forward primer** | **Reverse primer** |
| --- | --- | --- | --- | --- |
| D_comp1058_c0_seq1:1573 | C | G | GGAGGTCATCATACAAGGTTA | GAGGCTCGAGCTAACCAACC |
| D_comp19374_c0_seq1:702 | T | C | GCTGCACCATCTCCTATCAC | CCTTGAGTGCGCAGATTATG |
| B_comp2391_c0_seq1:284 | C | T | GAGACGAGCAACAGCATGC | GGAAACGGTATCAAGACATATGT |

**Table S1.** Marker variation screened across WAGTAIL accessions for marker validation. Primer sequences shown proved effective in screening targeted variation.
